# miRNA标志物对肺癌远处转移的预测价值

**DOI:** 10.3779/j.issn.1009-3419.2024.102.43

**Published:** 2024-12-20

**Authors:** Jingjing CONG, Anna WANG, Yingjia WANG, Xinge LI, Junjian PI, Kaijing LIU, Hongjie ZHANG, Xiaoyan YAN, Hongmei LI

**Affiliations:** ^1^266000 青岛，青岛大学附属医院肿瘤科; ^1^Department of Oncology, The Affiliated Hospital of Qingdao University, Qingdao 266000, China; ^2^250117 济南，山东第一医科大学基础医学院; ^2^College of Basic Medical Sciences, Shandong First Medical University, Jinan 250117, China; ^3^570000 海口，海南科技职业大学医药学院; ^3^College of Medicine, Hainan Vocational University of Scienceand Technology, Haikou 570000, China; ^4^266071 青岛，青岛大学青岛医学院; ^4^Qingdao Medical College, Qingdao University, Qingdao 266071, China

**Keywords:** 肺腺癌, 远处转移, miRNA标志物, 预测效能, 富集分析, Lung adenocarcinoma, Distant metastasis, miRNA signature, Predictive efficacy, Enrichment analysis

## Abstract

**背景与目的:**

肺癌是全球癌症相关死亡的主要原因，非小细胞肺癌（non-small cell lung cancer, NSCLC）是最常见的肺癌亚型。一半以上的NSCLC患者在诊断时已经发生转移，预后差。因此，有必要寻找新的生物标志物，用于预测NSCLC远处转移（distant metastasis, DM），以便指导后续治疗，从而改善NSCLC患者的预后。大量研究已经证实，微小RNAs（microRNAs, miRNAs）在肺癌组织中异常表达，对肿瘤的发生、进展起着重要作用。本研究的目的是鉴定DM和无远处转移（non-distant metastasis, NDM）的肺腺癌组织中差异表达的miRNAs，构建预测肺腺癌DM的miRNA标志物。

**方法:**

从癌症基因组图谱（The Cancer Genome Atlas, TCGA）数据库中下载肺腺癌患者的miRNAs表达数据及临床信息。应用生物信息学方法分析数据，包括R语言中的edgeR包、Kaplan-Meier曲线、受试者工作特征（receiver operating characteristic, ROC）曲线以及多种在线分析工具等。

**结果:**

DM组与NDM组间共鉴定出12个差异表达的miRNAs，筛选出8个miRNAs用于构建miRNAs标志物（miR-377-5p、miR-381-5p、miR-490-5p、miR-519d-5p、miR-3136-5p、miR-320e、miR-2355-5p、miR-6784-5p）。该miRNAs标志物预测DM的效能良好，ROC曲线下面积（area under the curve, AUC）为0.831。Logistic回归分析显示此miRNAs标志物是肺腺癌DM的独立危险因素。接下来，预测了8个miRNAs的靶基因，富集分析显示这些靶基因富集在多种通路，包括肿瘤通路、单纯疱疹病毒I型感染通路、PI3K-Akt通路、MAPK通路、Ras通路等。

**结论:**

此miRNAs标志物预测肺腺癌DM效能良好，有望成为肺腺癌DM的预测指标。

## Abstract (en)

**Background and objective:**

Lung cancer represents the main cause of cancer-related deaths worldwide, and non-small cell lung cancer (NSCLC) is the most main subtype. More than half of NSCLC patients have already developed distant metastasis (DM) at the time of diagnosis and have a poor prognosis. Therefore, it is necessary to find new biomarkers for predicting NSCLC DM in order to guide subsequent treatment and thus improve the prognosis of NSCLC patients. Numerous studies have shown that microRNAs (miRNAs) are abnormally expressed in lung cancer tissues and play an important role in tumorigenesis and progression. The aim of this study is to identify differentially expressed miRNAs in lung adenocarcinoma tissues with DM group compared to those with non-distant metastasis (NDM) group, and to construct a miRNA signature for predicting DM of lung adenocarcinoma.

**Methods:**

We first obtained miRNA and clinical data for patients with lung adenocarcinoma from The Cancer Genome Atlas (TCGA) database. Subsequently, bioinformatics analysis, which included different R packages, Kaplan-Meier analysis, receiver operating characteristic (ROC) curve, and a range of online analysis tools, was performed to analyze the data.

**Results:**

A total of 12 differentially expressed miRNAs were identified between the DM and NDM groups, and 8 miRNAs (miR-377-5p, miR-381-5p, miR-490-5p, miR-519d-5p, miR-3136-5p, miR-320e, miR-2355-5p, miR-6784-5p) were screened for constructing a miRNA signature. The efficacy of this miRNA signature in predicting DM was good with an area under the curve (AUC) of 0.831. Logistic regression analysis showed that this miRNA signature was an independent risk factor for DM of lung adenocarcinoma. Next, target genes of the eight miRNAs were predicted, and enrichment analysis showed that these target genes were enriched in a variety of pathways, including pathways in cancer, herpes simplex virus I infection, PI3K-Akt pathway, MAPK pathway, Ras pathway, etc.

**Conclusion:**

This miRNA signature has good efficacy in predicting DM of lung adenocarcinoma and has the potential to be a predictor of DM of lung adenocarcinoma.

肺癌是全球癌症相关死亡的主要原因，每年约有179万例死亡，肺癌分为非小细胞肺癌（non-small cell lung cancer, NSCLC）和小细胞肺癌（small cell lung cancer, SCLC），其中腺癌是NSCLC最常见的组织学亚型^[1]^。组织学亚型的比例因人种而异，在西方国家患者中腺癌占NSCLC的47%，而在中国患者中腺癌占55%-60%^[2]^。NSCLC患者的生存期取决于诊断时的分期，韩国的一项研究^[3]^显示，NSCLC的5年生存率I期为82%，II期为59%，III期为16%，IV期为10%。由此可见，NSCLC患者发生远处转移（distant metastasis, DM）后，患者的生存率明显下降。但是由于NSCLC早期无特异性症状及缺乏早期诊断方法，一半以上的患者在诊断时已经发生转移，总体中位生存期仅为7-12个月^[4]^。目前，根治性手术切除仍是I、II期和部分IIIA期NSCLC患者的标准疗法，然而，即使进行了根治性手术，部分患者仍然会出现转移，导致治疗失败^[5]^。因此，有必要寻找新的生物标志物，用于预测NSCLC DM，以便指导后续治疗，从而改善NSCLC患者的预后。

微小RNAs（microRNAs, miRNAs）是由19-24个核苷酸组成的非编码短链RNA，是在1993年由Lee等^[6]^在秀丽隐杆线虫中发现的，其通常通过与靶信使RNA的3´-非翻译区结合，通过调节信使RNA的稳定性或诱导其降解，而在转录后水平负性调节基因表达^[7,8]^。但是，在特定条件下，miRNAs也可促进靶信使RNA的翻译^[9]^。miRNAs仅由约3%的人类基因编码，但可以调控约30%的蛋白质编码基因，因此，它们可以调节包括代谢、生长、发育、免疫等在内的多种生物学功能^[10]^。多项研究^[11⇓-13]^已经证实，miRNAs在NSCLC的发生进展中起着重要作用，如miRNA-98-5p可通过靶向TGFBR1抑制NSCLC的增殖和转移^[11]^，miRNA-330-3p通过GRIA3促进NSCLC脑转移和上皮间充质转化^[12]^，miRNA-448可通过调控IRS2抑制NSCLC的进展^[13]^。先前的研究^[14]^表明，miRNAs的表达模式可用于癌症的早期诊断，还可用于预测癌症患者的预后^[15]^。因此，miRNAs作为诊断及预后生物标志物具有重大的潜力。

癌症基因组图谱（The Cancer Genome Atlas, TCGA）是一个公共数据库，提供癌症基因组数据，通过大规模基因组测序和综合分析，发现主要致癌基因组变异，从而用于改善癌症诊断、治疗和预防^[16]^。本研究的目的是通过分析TCGA数据库中的miRNAs数据，筛选DM和无远处转移（non-distant metastasis, NDM）的肺腺癌组织中差异表达的miRNAs，建立用于预测肺腺癌DM的miRNAs标志物，并预测其靶基因及其通路和功能，这可能为了解肺腺癌DM的潜在分子机制提供新的见解。

## 1 资料与方法

### 1.1 数据下载及处理

从TCGA数据库下载肺腺癌患者的miRNAs表达数据及临床信息（https://portal.gdc.cancer.gov/）。纳入标准：（1）样本包含miRNAs表达数据及临床信息；（2）样本具有预后信息；（3）患者之前均未接受过治疗。排除标准：样本分期不明确及总生存期为0。最后，共344例肺腺癌样本纳入研究，其中IV期23例，为DM组，I-III期321例，为NDM组。

### 1.2 差异表达miRNAs的筛选及miRNAs标志物的构建

使用R语言（4.3.1版）对下载的数据进行分析并绘图。应用“edgeR”包对DM组和NDM组的miRNAs表达数据进行差异分析，计算每个miRNAs的倍数变化（fold change, FC），以|log2 FC|>1和错误发现率（false discovery rate, FDR）<0.05为标准，鉴定差异表达miRNAs。通过最小绝对值收敛和选择算子（least absolute shrinkage and selection operator, LASSO）回归，从差异表达miRNAs中筛选关键的miRNAs，构建预测肺腺癌DM的miRNAs标志物，利用回归系数和miRNA的相应百万读数（reads per million, RPM）值计算所有患者的风险评分（miRNAs标志物：风险评分=β_1_×miRNA_1_EXP+β_2_×miRNA_2_EXP+...+β_n_×miRNA_n_EXP。β为相应miRNAs的回归系数，miRNA_n_EXP为相应miRNAs的表达量）。

### 1.3 靶基因预测及富集分析

使用miRWalk和TargetScan数据库预测miRNAs的靶基因，并利用韦恩图鉴定重叠的靶基因。然后，利用注释、可视化和集成发现的数据库（Database for Annotation, Visualization and Integrated Discovery, DAVID）线上分析工具对靶基因进行基因本体（Gene Ontology, GO）和京都基因和基因组百科全书（Kyoto Encyclopedia of Genes and Genomes, KEGG）富集分析。

### 1.4 构建蛋白质相互作用（protein-protein interaction, PPI）网络并筛选核心基因

应用Cytoscape软件的cytoHubba插件筛选核心基因，并使用stringAPP插件，构建核心基因PPI网络，并对网络进行可视化。运用微生信平台构建miRNAs与核心基因的关系图。

### 1.5 统计学方法

采用R（4.3.1版）软件处理数据及生成图像。分类变量用n（%）表示，采用χ^2^检验进行比较。连续变量用均数±标准差表示，采用t检验进行比较。LASSO回归用于筛选关键miRNAs。受试者工作特征（receiver operating characteristic, ROC）曲线用于验证miRNAs标志物的预测性能。Kaplan-Meier法用于评估生存情况，Log-rank检验用于组间比较。利用单变量和多变量Logistic回归分析探讨影响肺腺癌DM的独立危险因素。单变量分析中，*P*<0.1的变量纳入多变量分析。*P*<0.05为具有统计学差异。

## 2 结果

### 2.1 患者的基线特征

本研究的流程图见图1。从TCGA数据库中下载513例肺腺癌患者的miRNAs测序数据，排除分期不明确及总生存期为0的患者，共344例肺腺癌患者纳入研究，其中DM组23例、NDM组321例，每个样本检测2213种miRNAs，排除表达量极低的miRNAs（80%的样本中表达量为0），使用剩余545种miRNAs测序数据的RPM值进行后续分析。并从TCGA数据库中下载患者的临床信息。患者临床信息见表1，DM组和NDM组的基线特征均衡。

**图 1:**
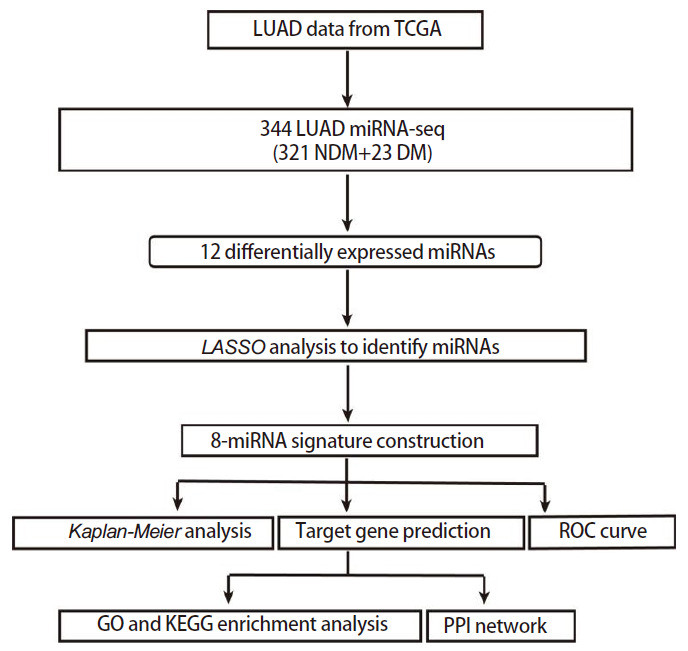
本研究流程图

**表 1:** 研究样本临床特征

Characterics	NDM group (*n*=321)	DM group (*n*=23)	P
Age (Mean±SD, yr)	65.1±9.8	61.2±12.1	0.145
Gender			0.372
Male	157 (48.91%)	14 (60.92%)	
Female	164 (51.09%)	9 (39.08%)	
Smoking			0.999
No	98 (30.52%)	7 (30.41%)	
Yes	223 (69.48%)	16 (69.59%)	
T stage			0.103
T1-T2	281 (87.51%)	17 (73.92%)	
T3-T4	40 (12.49%)	6 (26.08%)	
N stage			0.068
N0-N1	274 (85.40%)	16 (69.61%)	
N2-N3	47 (14.60%)	7 (30.39%)	

SD: standard deviation.

### 2.2 差异表达miRNAs的筛选

应用“edgeR”包对DM组和NDM组的miRNAs表达数据进行差异分析，共鉴定出12个差异表达的miRNAs，且均在DM组中上调（图2）。

**图 2:**
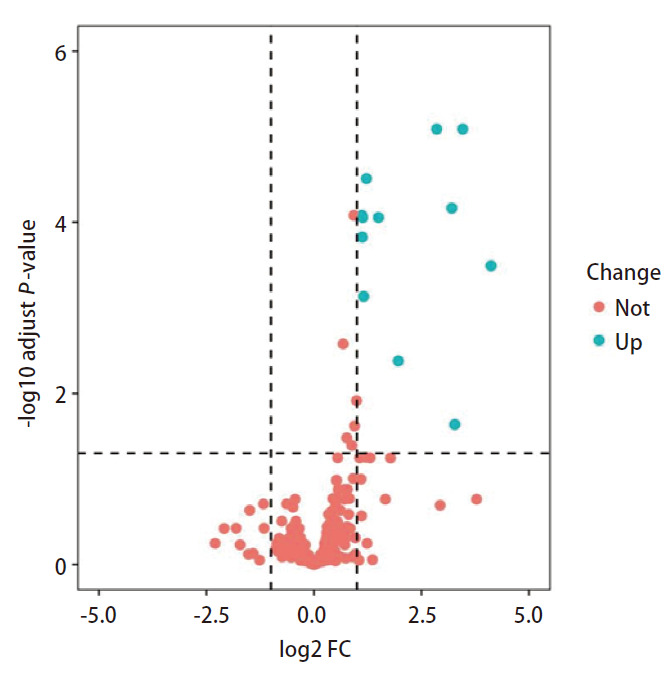
DM组和NDM组差异表达miRNAs的火山图

### 2.3 miRNAs标志物的构建

对12个miRNAs进行LASSO回归分析，鉴定出8个关键的miRNAs用于构建miRNAs标志物（图3）。这8个miRNAs包括miR-377-5p、miR-381-5p、miR-490-5p、miR-519d-5p、miR-3136-5p、miR-320e、miR-2355-5p、miR-6784-5p。并计算每位患者的风险评分，miRNAs标志物如下所示：风险评分=（0.000219684×miR-377-5pRPM）+（0.00000418×miR-381-5pRPM）+（0.00038118×miR-490-5pRPM）+（0.000980534×miR-519d-5pRPM）+（0.056667066×miR-3136-5pRPM）+（0.032313511×miR-320eRPM）+（0.049995949×miR-2355-5pRPM）+（0.0931777×miR-6784-5pRPM）。

**图 3:**
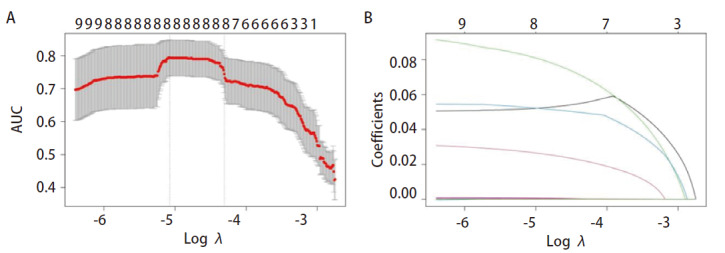
miRNAs标志物的构建。A：在LASSO分析中通过10倍交叉验证选择参数λ；B：12个差异表达miRNAs的LASSO回归系数谱。

### 2.4 miRNAs标志物对肺腺癌DM的预测价值

通过绘制ROC曲线评估miRNAs标志物对肺腺癌DM的预测价值，miRNAs标志物的曲线下面积（area under the curve, AUC）为0.831（95%CI: 0.730-0.933, *P*<0.0001），miR-377-5p、miR-381-5p、miR-490-5p、miR-519d-5p、miR-3136-5p、miR-320e、miR-2355-5p、miR-6784-5p的AUC分别为0.558（95%CI: 0.426-0.690, *P*=0.165）、0.593（95%CI: 0.457-0.729, *P*=0.068）、0.565（95%CI: 0.448-0.682, *P*=0.15）、0.582（95%CI: 0.443-0.721, *P*=0.094）、0.425（95%CI: 0.286-0.565, *P*=0.116）、0.510（95%CI: 0.367-0.652, *P*=0.438）、0.523（95%CI: 0.380-0.665, *P*=0.356）、0.691（95%CI: 0.583-0.799, *P*=0.001），表明与上述8种miRNAs相比，我们构建的miRNAs标志物对肺腺癌DM的预测能力更好（图4A-4I）。确定miRNAs标志物ROC曲线的最佳截断值为1.944，以1.944为截断点，将344例样本分为高风险组（≥1.944, *n*=284）和低风险组（<1.944, *n*=60），运用Kaplan-Meier方法进行生存分析，结果表明，高风险组较低风险组的预后更差（*P*=0.003）（图4J）。

**图 4:**
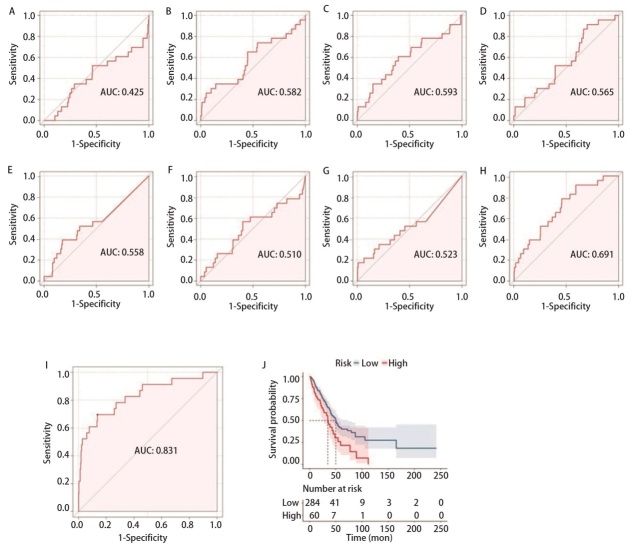
miRNAs标志物的预测价值。miR-3136-5p（A）、miR-519d-5p（B）、miR-381-5p（C）、miR-490-5p（D）、miR-377-5p（E）、miR-320e（F）、miR-2355-5p（G）、miR-6784-5p（H）和miRNA标志物（I）预测肺腺癌远处转移的ROC曲线；J：miRNAs标志物的Kaplan-Meier曲线。

将miRNAs标志物与临床特征（年龄、性别、吸烟史、T分期、N分期）进行Logistic回归分析，验证构建的miRNAs标志物对转移的影响，在单变量分析中，年龄（OR=0.96, *P*=0.075）、T分期（OR=2.48, *P*=0.072）、N分期（OR=2.55, *P*=0.051）和miRNAs标志物（OR=14.39, *P*<0.001）的*P*<0.1，纳入多变量分析。多变量Logistic分析显示，miRNAs标志物（OR=15.62, *P*<0.001）是肺腺癌DM的独立危险因素（表2）。

**表 2:** Logistic回归分析

Characterics	Univariate analysis			Multivariate analysis	
	OR (95%CI)	P		OR (95%CI)	P
Age	0.96 (0.92-1.00)	0.075		0.97 (0.93-1.02)	0.249
Gender (Male vs Female)	0.62 (0.25-1.44)	0.272			
Smoking (No vs Yes)	1.00 (0.41-2.68)	0.992			
T stage (T1-2 vs T3-4)	2.48 (0.85-6.37)	0.072		2.38 (0.69-7.53)	0.150
N stage (N0-1 vs N2-3)	2.55 (0.94-6.33)	0.051		2.81 (0.88-8.54)	0.070
miRNAs signature (Low risk vs High risk)	14.39 (5.80-39.31)	<0.001		15.62 (6.04-45.03)	<0.001

OR: odds ratio; CI: confidence interval.

### 2.5 靶基因预测及富集分析

使用miRWalk和TargetScan数据库预测上述8个miRNAs的靶基因。miR-377-5p有1021个重叠靶基因，miR-381-5p有174个重叠靶基因，miR-490-5p有690个重叠靶基因，miR-519d-5p有646个重叠靶基因，miR-3136-5p有585个重叠靶基因，miR-320e有602个重叠靶基因，miR-2355-5p有807个重叠靶基因，miR-6784-5p有444个重叠靶基因（图5）。8个miRNAs共有3664个靶基因。然后，对3664个靶基因进行富集分析以明确靶基因的生物学功能。

**图 5:**
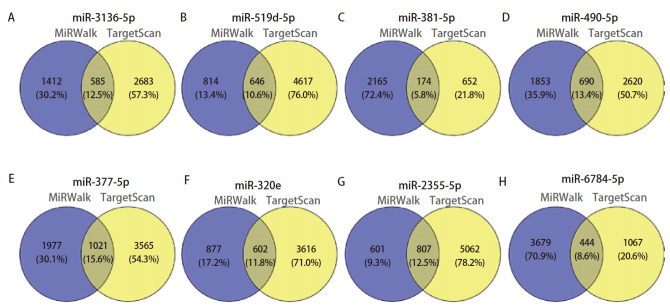
8个miRNAs重叠靶基因的韦恩图。A：miR-3136-5p；B：miR-519d-5p；C：miR-381-5p；D：miR-490-5p；E：miR-377-5p；F：miR-320e；G：miR-2355-5p；H：miR-6784-5p。

使用DAVID线上分析工具对3664个靶基因进行GO和KEGG富集分析。如图6所示，生物学过程（biological process, BP）分析显示，这些靶基因主要参与了转录调控、RNA聚合酶II启动子转录调控、细胞内信号转导、细胞增殖正调控、神经系统发育、蛋白磷酸化等过程。细胞成分（cellular component, CC）分析结果表明，这些靶基因主要富集在细胞核、细胞质和核质中。分子功能（molecular function, MF）分析显示，靶基因主要富集于蛋白结合、金属离子结合、RNA聚合酶II转录因子活性序列特异性DNA结合、RNA聚合酶II核心启动子近端区域序列特异性DNA结合和转录因子活性序列特异性DNA结合等。KEGG通路分析结果显示，靶基因在肿瘤通路、单纯疱疹病毒I型感染通路、磷脂酰肌醇-3-激酶（phosphoinositide 3-kinase, PI3K）-蛋白激酶B（protein kinase B, Akt）通路、丝裂原活化蛋白激酶（mitogen-activated protein kinase, MAPK）通路、大鼠肉瘤（rat sarcoma, Ras）通路人巨细胞病毒感染、钙信号通路、蛋白聚糖、内吞作用、黏着、cAMP信号通路中明显富集，而这些通路通常与肿瘤进展相关，这表明我们筛选的miRNAs在肺腺癌转移中发挥了潜在的作用（图7）。

**图 6:**
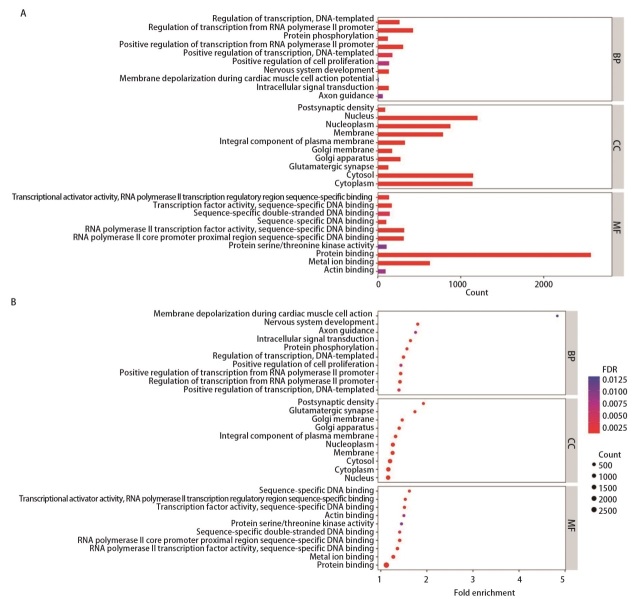
重叠靶基因的GO分析图。A：条形图；B：气泡图。

**图 7:**
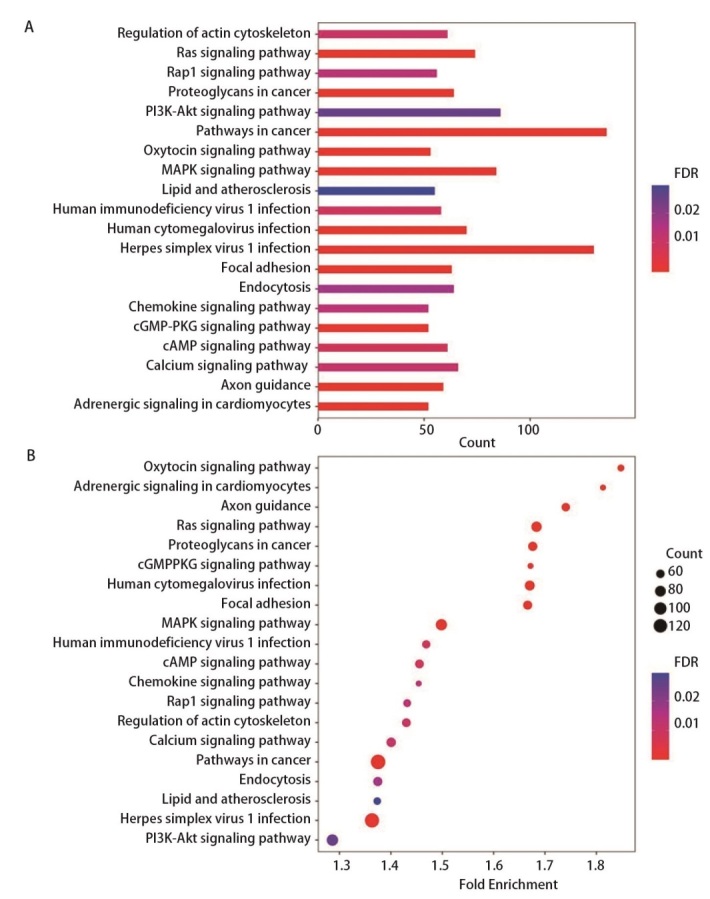
KEGG分析图。A：条形图；B：气泡图。

### 2.6 PPI网络构建及核心基因筛选

利用Cytoscape软件的cytoHubba插件筛选出24个核心基因，并使用stringAPP插件，构建核心基因的PPI网络（表3、图8A），其中miR-377-5p与10个核心基因（RPF2、RBM28、UTP6、KRR1、GNL3L、LSG1、UTP23、RRP8、NLE1、RBM34）相关、miR-490-5p与3个核心基因（BMS1、RBM28、NLE1）相关、miR-519d-5p与4个核心基因（RPF2、WDR12、RSL24D1、RCL1）相关、miR-320e与4个核心基因（RBM28、KRR1、DDX17、DNTTIP2）相关、miR-2355-5p与4个核心基因（BMS1、NOP9、KRR1、DDX51）相关、miR-6784-5p与5个核心基因（NAT10、DDX31、RRP15、SURF6、POLR1A）相关，miR-381-5p与1个核心基因（DNTTIP2）相关，miR-3136-5p与1个核心基因（PNO1）相关（图8B）。

**表 3:** 8个miRNAs的24个核心基因

Gene symbol	Score
NAT10	7.63×10^15^
BMS1	7.63×10^15^
RPF2	7.63×10^15^
RBM28	7.63×10^15^
UTP6	7.63×10^15^
WDR12	7.63×10^15^
NOP9	7.63×10^15^
KRR1	7.63×10^15^
GNL3L	7.63×10^15^
RSL24D1	7.63×10^15^
DDX51	7.62×10^15^
DDX31	7.60×10^15^
PNO1	7.60×10^15^
LSG1	7.57×10^15^
UTP23	7.56×10^15^
RRP15	6.83×10^15^
RRP8	6.80×10^15^
SURF6	6.47×10^15^
NLE1	7.80×10^14^
DDX17	7.32×10^14^
POLR1A	6.57×10^13^
RCL1	4.48×10^13^
RBM34	2.64×10^13^
DNTTIP2	2.25×10^13^

**图 8:**
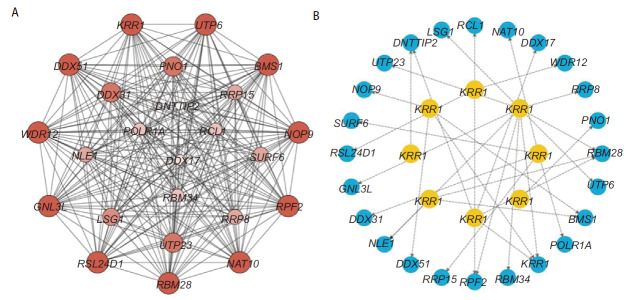
网络图。A：24个核心基因的PPI网络图；B：miRNAs和核心基因关系图。

## 3 讨论

肺癌是癌症相关死亡的主要原因，仅有21.7%的患者生存期超过5年^[17]^，肺癌包括NSCLC和SCLC，其中腺癌是NSCLC中最常见的组织学类型。由于NSCLC在早期临床症状不明显，一半以上的患者在诊断时已经转移，5年生存率仍然很低^[18]^。因此有必要寻找能有效预测NSCLC DM的生物标志物，从而改善患者的生存。miRNAs是一种短链非编码RNA，在多种恶性肿瘤的进展中发挥重要作用，包括NSCLC。多种miRNAs已被证实可作为NSCLC的生物标志物^[19⇓-21]^。且在多项研究中，构建的miRNAs模型有作为NSCLC早期诊断及预测预后的生物标志物的潜力。Zhou等^[22]^研究显示，血清miR-942和miR-601在NSCLC组织中表达显著上调，且在NSCLC的早期诊断中表现出比癌胚抗原（carcinoembryonic antigen, CEA）、细胞角蛋白19片段（cytokeratin 19 fragment 21-1, CYFRA21-1）和鳞状细胞癌相关抗原（squamous cell carcinoma associated antigen, SCCA）更好的性能，联合检测血清miR-942和miR-601可提高NSCLC早期诊断的准确性，此外，血清中miR-942和miR-601高表达均与不良预后有关，是NSCLC的独立预后因素。在另一项研究^[23]^中，构建的miRNAs模型可用于预测肺鳞癌的预后。在一项包含334例IV期NSCLC患者的研究^[24]^中，包含5个miRNAs的风险评分模型，可以用于预测免疫治疗后的总生存率，且其性能优于组织程序性细胞死亡配体1（programmed cell death ligand 1, PD-L1）检测。

在本研究中，我们共鉴定出12个差异表达的miRNAs，并筛选出8个关键miRNAs，构建了miRNAs标志物，且该标志物预测肺腺癌DM的效能良好（AUC为0.831），并发现其是肺腺癌DM的独立危险因素。研究结果表明，我们构建的miRNAs标志物，有预测肺腺癌DM的潜力。

Chen等^[25]^建立了一个肺腺癌预后相关的miRNAs标志物，研究显示，此miRNAs标志物是肺腺癌患者的独立预后因素。而我们构建的miRNAs标志物是肺腺癌患者DM的独立危险因素。有研究^[26]^发现，肿瘤大小是肺腺癌发生DM的重要预测因素，而且淋巴结转移可促进NSCLC DM^[27]^。本研究Logistic分析中T分期和N分期并未显示出是肺腺癌DM的独立预测因素，我们考虑到不能排除因DM组样本量较少，导致了统计学偏倚所致。

以往研究发现，这8个miRNAs在不同肿瘤的诊断、治疗和进展中发挥着重要作用。Wu等^[28]^研究发现，miR-377-5p在肺癌组织及细胞系中下调，其可通过靶向AKT1抑制细胞活力、增殖、迁移、侵袭和诱导细胞周期停滞。另一项关于宫颈癌的研究^[29]^显示，miR-377-5p低表达与宫颈癌较差的预后相关，这可能意味着miR-377-5p对肿瘤进展可能发挥抑制作用。代谢综合征（metabolic syndrome, MeS）是一组病理生理学疾病，包括以下3或3种以上因素：血压≥130/85 mmHg、甘油三酯≥150 mg/dL、腹部肥胖、高密度脂蛋白<50 mg/dL、空腹血糖≥110 mg/dL，MeS是乳腺癌的危险因素，与高级别肿瘤、转移和复发有关，而Farré等^[30]^研究发现MeS可诱导miR-381-5p和miR-194-1-5p表达，表明MeS通过调节各种基因和miRNAs来调控乳腺癌的进展，这可能意味着miR-381-5p可能促进乳腺癌的进展。多项研究显示，在胃癌^[31]^、肾细胞癌^[32]^、肝细胞癌^[33]^、结肠癌^[34]^、喉癌^[35]^、膀胱癌^[36]^等恶性肿瘤中，miR-490-5p抑制肿瘤进展。然而，另一项研究^[37]^显示，miR-490家族中的另一个成员miR-490-3p却促进乳腺浸润性导管癌转移进展。Ye等^[38]^发现，circ_0007385表达在NSCLC肿瘤组织尤其是晚期肿瘤组织中上调，且miR-519d-3p的表达与circ_0007385的表达呈负相关，circ_0007385敲低可抑制NSCLC细胞的增殖、迁移和侵袭能力，而沉默miR-519d-3p可逆转circ_0007385敲低所产生的抑制作用，此研究表明miR-519d-3p对NSCLC进展发挥抑制作用。在另一项研究^[39]^中，乳腺癌组织中miR-519d-5p的表达降低，且miR-519d-5p过表达可以增加细胞对顺铂的敏感性。Liu等^[40]^通过分析来自46个正常样本和513个肺腺癌组织样本的miRNAs测序数据，鉴定出4种miRNAs，这4种miRNAs（miR-1246、miR-9-5p、miR-31-3p、miR-3136-5p）在肿瘤组织中均上调，并构建了4-miRNA风险评分模型，高风险组较低风险组预后更差。一项关于结直肠癌的研究^[41]^显示，在III期结直肠癌中miR-320e表达水平显著升高，且miR-320e表达增加与较差的无病生存期有关，表明miR-320e可作为III期结直肠癌患者预后不良的生物标志物。而miR-320家族中的另一成员miR-320a具有不同的功能，Sun等^[42]^研究发现miR-320a在结肠癌细胞系和组织中下调，并可抑制结肠癌细胞的生长。一项关于食管鳞状细胞癌的研究^[43]^显示，miR-2355-5p可通过SOCS2/JAK2/Stat5信号通路促进食管鳞状细胞癌的生长和侵袭。然而，Yu等^[44]^研究证实miR-2355-5p对三阴性乳腺癌发挥抑制作用。关于miR-6784-5p，已被证实可抑制皮肤鳞状细胞癌进展^[45]^。通过以上研究发现，在不同种类的癌症以及同一种癌症的不同阶段，miRNAs可能有多个不同的靶点。由此可见，miRNAs的功能很复杂，可能随癌症类型和癌症阶段的不同而变化，既可能是致癌因子，也可能是抑癌基因。因此，这8种miRNAs在肺腺癌DM中的作用还有待进一步研究。

为了深入了解这8个miRNAs在肺腺癌DM中的潜在机制，我们还利用生物信息学方法预测了这8个miRNAs的靶基因，并对靶基因进行了GO和KEGG富集分析，包括生物学过程、细胞组分、分子功能和KEGG通路。KEGG分析显示，这些靶基因主要在癌症通路、单纯疱疹病毒I型感染、PI3K/Akt通路、MAPK通路、Ras通路、人类巨细胞病毒感染、钙通路、癌症中的蛋白多糖和内吞作用等通路中富集。有研究^[46,47]^发现，PI3K/Akt通路的异常激活可促进NSCLC肺和脑转移。同样，NF-κB/MAPK通路的异常激活也与癌症进展相关。有研究^[48,49]^证实，NF-κB/MAPK通路的激活可促进NSCLC的进展。Tian等^[50]^研究发现miR-135a通过抑制RAS通路可抑制NSCLC的进展。另外的一项研究^[51]^显示，RAS/MEK/ERK和PI3K/Akt信号通路被抑制后，KRAS突变型NSCLC细胞的增殖和转移也被抑制。这些研究表明，这8种miRNAs的下游靶基因参与了多种生物学过程，进一步表明，这8种miRNAs在肺腺癌DM中发挥了关键作用。

最后，我们又对上述靶基因进行了PPI网络构建，筛选出前24个核心基因（NAT10、BMS1、RPF2、RBM28、UTP6、WDR12、NOP9、KRR1、GNL3L、RSL24D1、DDX51、DDX31、PNO1、LSG1、UTP23、RRP15、RRP8、SURF6、NLE1、DDX17、POLR1A、RCL1、RBM34、DNTTIP2）。这些核心基因对癌症的发生进展起着重要作用。NAT10是一种核仁乙酰转移酶，可促进各种癌症的发生发展，研究^[52]^发现，NAT10在NSCLC组织中显著过表达，并促进NSCLC发展。RPF2参与核糖体的生物发生，而核糖体生物发生失调会增加患癌风险，有研究^[53]^发现，RPF2过表达可促进肝细胞癌细胞增殖、迁移和侵袭。RBM蛋白家族在肝细胞癌的发展中发挥着重要作用，Wu等^[54]^构建了包括RBM8A、RBM19、RBM28和RBM45在内的4个基因的预后模型，高危患者预后更差，此外，RBM45敲低可抑制肝细胞癌细胞增殖，表明RBM家族成员可促进肝细胞癌进展。另有研究^[55]^发现RBM34过表达可促进肝癌进展并与预后不良相关。WDR12在核糖体生物合成途径中起着重要作用，Yin等^[56]^研究证实，WDR12的表达上调与肝细胞癌患者的总生存期短显著相关，WDR12敲低可抑制肝细胞癌细胞增殖和迁移，表明WDR12可促进肝细胞癌进展。GNL3L在多种类型的癌症中上调，有研究^[57]^显示，GNL3L在食管癌组织中表达上调，且GNL3L高表达会促进食管癌的进展，并与患者的不良预后有关。有研究^[58]^发现，DDX51在食管鳞癌组织中上调，且高表达患者预后不良，敲低DDX51可抑制食管鳞癌细胞增殖并促进其凋亡，表明DDX51可促进食管鳞状细胞癌进展。DDX31是一种在绝大多数肾细胞癌中上调的核仁蛋白，DDX31的敲低显著抑制肾细胞癌细胞生长，DDX31过表达则促进肾细胞癌细胞增殖^[59]^。PNO1是一种重要的核糖体，而核糖体的激活会促进癌症的发展，Han等^[60]^研究发现，PNO1在肝细胞癌组织中上调，且PNO1过表达可促进肝细胞癌细胞增殖，并抑制凋亡。UTP23也是一种核仁蛋白，对核糖体的生物发生至关重要，有研究^[61]^发现，乳腺癌组织中UTP23表达上调，并且UTP23高表达与预后差有关，且UTP23敲低可抑制乳腺癌细胞增殖。Zhao等^[62]^研究发现，RRP15在肝细胞癌组织中表达上调，且与不良预后有关，RRP15敲低可抑制肝细胞癌细胞增殖和生长。据报道DDX17具有致癌作用，在有肝外转移的原发性肝细胞癌组织中，DDX17表达上调，且与不良预后密切关联，体内和体外实验证实DDX17可促进肝细胞癌细胞生长和转移^[63]^。有些基因在肺腺癌中的研究尚不深入，这24个核心基因可以为肺腺癌的进一步研究提供方向。

基于上述分析，我们认为此miRNAs标志物可以作为新的生物标志物用于预测肺腺癌DM。本研究对于更好地了解肺腺癌DM具有重要意义。

然而，本研究也存在一定的局限性。首先，我们的数据来自TCGA数据库，研究结果可能存在偏倚。其次，我们分析了DM组和NDM组的miRNAs表达数据，但在不同阶段、性别、年龄和吸烟史的肺腺癌患者中，miRNAs表达水平可能有所不同，因此研究结果可能存在偏差。最后，研究结果并未在临床样本中进行验证。在后续研究中，我们将收集样本验证此miRNAs标志物对肺腺癌DM的预测价值，并进一步研究此miRNAs标志物在肺腺癌DM中的作用机制。

## References

[1] Thai AA, Solomon BJ, Sequist LV, et al. Lung cancer. Lancet, 2021, 398(10299): 535-554. doi: 10.1016/S0140-6736(21)00312-3 34273294

[2] Chen P, Liu Y, Wen Y, et al. Non-small cell lung cancer in China. Cancer Commun (Lond), 2022, 42(10): 937-970. doi: 10.1002/cac2.12359 36075878 PMC9558689

[3] Jeon DS, Kim HC, Kim SH, et al. Five-year overall survival and prognostic factors in patients with lung cancer: Results from the Korean Association of Lung Cancer Registry (KALC-R) 2015. Cancer Res Treat, 2023, 55(1): 103-111. doi: 10.4143/crt.2022.264 35790197 PMC9873320

[4] Yang Z, Wang H, Zhao Z, et al. Gene-microRNA network analysis identified seven hub genes in association with progression and prognosis in non-small cell lung cancer. Genes, 2022, 13(8): 1480. doi: 10.3390/genes13081480 PMC9407881 36011391

[5] Liu XP, Li X, Yang F. Pattern of recurrence and metastasis after radical resection of non-small cell lung cancer. Zhongguo Feiai Zazhi, 2022, 25(1): 26-33. 35078282 10.3779/j.issn.1009-3419.2021.102.50 PMC8796126

[6] Lee RC, Feinbaum RL, Ambros V. The C. elegans heterochronic gene lin-4 encodes small RNAs with antisense complementarity to lin-14. Cell, 1993, 75(5): 843-854. doi: 10.1016/0092-8674(93)90529-y 8252621

[7] Ravegnini G, Gorini F, De Crescenzo E, et al. Can miRNAs be useful biomarkers in improving prognostic stratification in endometrial cancer patients? An update review. Int J Cancer, 2022, 150(7): 1077-1090. doi: 10.1002/ijc.33857 34706070 PMC9298718

[8] Khan P, Ebenezer NS, Siddiqui JA, et al. MicroRNA-1: Diverse role of a small player in multiple cancers. Semin Cell Dev Biol, 2022, 124: 114-126. doi: 10.1016/j.semcdb.2021.05.020 34034986 PMC8606619

[9] Marcuello M, Vymetalkova V, Neves RPL, et al. Circulating biomarkers for early detection and clinical management of colorectal cancer. Mol Aspects Med, 2019, 69: 107-122. doi: 10.1016/j.mam.2019.06.002 31189073

[10] Menon A, Abd-aziz N, Khalid K, et al. miRNA: A promising therapeutic target in cancer. Int J Mol Sci, 2022, 23(19): 11502. doi: 10.3390/ijms231911502 PMC9569513 36232799

[11] Jiang F, Yu Q, Chu Y, et al. MicroRNA-98-5p inhibits proliferation and metastasis in non-small cell lung cancer by targeting TGFBR1. Int J Oncol, 2019, 54(1): 128-138. doi: 10.3892/ijo.2018.4610 30387848 PMC6255066

[12] Wei C, Zhang R, Cai Q, et al. MicroRNA-330-3p promotes brain metastasis and epithelial-mesenchymal transition via GRIA 3 in non-small cell lung cancer. Aging, 2019, 11(17): 6734-6761. doi: 10.18632/aging.102201 31498117 PMC6756898

[13] Gao J, Feng X, Wang F, et al. microRNA-448 inhibits the progression of non-small-cell lung cancer through regulating IRS2. J Cell Biochem, 2019, 120(8): 13453-13463. doi: 10.1002/jcb.28619 30912183

[14] Chang YA, Weng SL, Yang SF, et al. A three-microRNA signature as a potential biomarker for the early detection of oral cancer. Int J Mol Sci, 2018, 19(3): 758. doi: 10.3390/ijms19030758 PMC5877619 29518940

[15] Li X, Shi Y, Yin Z, et al. An eight-miRNA signature as a potential biomarker for predicting survival in lung adenocarcinoma. J Transl Med, 2014, 12: 159. doi: 10.1186/1479-5876-12-159 24893932 PMC4062505

[16] Tomczak K, Czerwinska P, Wiznerowicz M. The Cancer Genome Atlas (TCGA): An immeasurable source of knowledge. Contemp Oncol (Pozn), 2015, 19(1A): A68-A77. doi: 10.5114/wo.2014.47136 PMC4322527 25691825

[17] Ettinger DS, Wood DE, Aisner DL, et al. Non-small cell lung cancer, version 3. 2022, NCCN Clinical Practice Guidelines in Oncology. J Natl Compr Canc Netw, 2022, 20(5): 497-530. doi: 10.6004/jnccn.2022.0025 35545176

[18] Lv J, An J, Zhang YD, et al. A three serum miRNA panel as diagnostic biomarkers of radiotherapy-related metastasis in non-small cell lung cancer. Oncol Lett, 2020, 20(5): 236. doi: 10.3892/ol.2020.12099 PMC7500041 32968458

[19] Zou Y, Jing C, Liu L, et al. Serum microRNA-135a as a diagnostic biomarker in non-small cell lung cancer. Medicine, 2019, 98(50): e17814. doi: 10.1097/MD.0000000000017814 PMC6922492 31852062

[20] Liu F, Li T, Hu P, et al. Upregulation of serum miR-629 predicts poor prognosis for non-small-cell lung cancer. Dis Markers, 2021, 2021: 8819934. doi: 10.1155/2021/8819934 33763157 PMC7946467

[21] Khandel WA, Sharma U, Barwal TS, et al. Circulating miR-320a acts as a tumor suppressor and prognostic factor in non-small cell lung cancer. Front Oncol, 2021, 11: 645475. doi: 10.3389/fonc.2021.645475 33833996 PMC8021852

[22] Zhou C, Chen Z, Zhao L, et al. A novel circulating miRNA-based signature for the early diagnosis and prognosis prediction of non-small-cell lung cancer. J Clin Lab Anal, 2020, 34(11): e23505. doi: 10.1002/jcla.23505 PMC7676218 33463758

[23] Liu H, Li T, Dong C, et al. Identification of miRNA signature for predicting the prognostic biomarker of squamous cell lung carcinoma. PLoS One, 2022, 17(3): e0264645. doi: 10.1371/journal.pone.0264645. PMC8923497 35290415

[24] Rajakumar T, Horos R, Jehn J, et al. A blood-based miRNA signature with prognostic value for overall survival in advanced stage non-small cell lung cancer treated with immunotherapy. NPJ Precis Oncol, 2022, 6(1): 19. doi: 10.1038/s41698-022-00262-y PMC8971493 35361874

[25] Chen B, Gao T, Yuan W, et al. Prognostic value of survival of microRNAs signatures in non-small cell lung cancer. J Cancer, 2019, 10(23): 5793-5804. doi: 10.7150/jca.30336 31737116 PMC6843868

[26] Soewondo W, Adzhani F, Hanafi M, et al. Lung adenocarcinoma size as a predictor of distant metastasis: A CT scan-based measurement. Narra J, 2024, 4(2): e1024. doi: 10.52225/narra.v4i2.1024 PMC11394171 39280288

[27] Zhang X, Ma L, Xue M, et al. Advances in lymphatic metastasis of non-small cell lung cancer. Cell Commun Signal, 2024, 22(1): 201. doi: 10.1186/s12964-024-01574-1 PMC10986052 38566083

[28] Wu H, Liu HY, Liu WJ, et al. miR-377-5p inhibits lung cancer cell proliferation, invasion, and cell cycle progression by targeting AKT1 signaling. J Cell Biochem, 2019, 120(5): 8120-8128. doi: 10.1002/jcb.28091 30485528

[29] Wang D, Zhang Y, Ren D, et al. Bioinformatics analysis illustrates the functions of miR-377-5p in cervical cancer. Biotechnol Genet Eng Rev, 2024, 40(4): 4238-4249. doi: 10.1080/02648725.2023.2208453 37144663

[30] Farre PL, Scalise GD, Duca RB, et al. CTBP1 and metabolic syndrome induce an mRNA and miRNA expression profile critical for breast cancer progression and metastasis. Oncotarget, 2018, 9(17): 13848-13858. doi: 10.18632/oncotarget.24486 29568399 PMC5862620

[31] Li J, Xu X, Liu C, et al. MiR-490-5p restrains progression of gastric cancer through DTL repression. Gastroenterol Res Pract, 2021, 2021: 2894117. doi: 10.1155/2021/2894117 34594374 PMC8478551

[32] Chen K, Zeng J, Tang K, et al. miR-490-5p suppresses tumour growth in renal cell carcinoma through targeting PIK3CA. Biol Cell, 2016, 108(2): 41-50. doi: 10.1111/boc.201500033 26559013 PMC4744944

[33] Chen W, Ye L, Wen D, et al. MiR-490-5p inhibits hepatocellular carcinoma cell proliferation, migration and invasion by directly regulating ROBO1. Pathol Oncol Res, 2019, 25(1): 1-9. doi: 10.1007/s12253-017-0305-4 28924964

[34] Yang YJ, Luo S, Xu ZL. Effects of miR-490-5p targeting CDK1 on proliferation and apoptosis of colon cancer cells via ERK signaling pathway. Eur Rev Med Pharmacol Sci, 2022, 26(6): 2049-2056. doi: 10.26355/eurrev_202203_28353 35363355

[35] Abdeyrim A, Cheng X, Lian M, et al. miR-490-5p regulates the proliferation, migration, invasion and epithelial-mesenchymal transition of pharyngolaryngeal cancer cells by targeting mitogen-activated protein kinase kinase kinase 9. Int J Mol Med, 2019, 44(1): 240-252. doi: 10.3892/ijmm.2019.4196 31115491 PMC6559303

[36] Li S, Xu X, Xu X, et al. MicroRNA-490-5p inhibits proliferation of bladder cancer by targeting c-Fos. Biochem Biophys Res Commun, 2013, 441(4): 976-981. doi: 10.1016/j.bbrc.2013.11.006 24220339

[37] Lu N, Zhang M, Lu L, et al. miRNA-490-3p promotes the metastatic progression of invasive ductal carcinoma. Oncol Rep, 2021, 45(2): 706-716. doi: 10.3892/or.2020.7880 33416185 PMC7757091

[38] Ye Y, Zhao L, Li Q, et al. circ_ 0007385 served as competing endogenous RNA for miR-519d-3p to suppress malignant behaviors and cisplatin resistance of non-small cell lung cancer cells. Thorac Cancer, 2020, 11(8): 2196-2208. doi: 10.1111/1759-7714.13527 32602212 PMC7396374

[39] Li D, Wang T, Yu Z, et al. MiR-519d-5p modulates the sensitivity of breast cancer to chemotherapy by forming a negative feedback loop with RELA. Ann Transl Med, 2021, 9(14): 1171. doi: 10.21037/atm-21-3241 PMC8350717 34430612

[40] Liu CH, Liu SH, Lai YL, et al. Using bioinformatics approaches to identify survival-related oncomiRs as potential targets of miRNA-based treatments for lung adenocarcinoma. Comput Struct Biotechnol J, 2022, 20: 4626-4635. doi: 10.1016/j.csbj.2022.08.042 36090818 PMC9449502

[41] Perez-carbonell L, Sinicrope FA, Alberts SR, et al. MiR-320e is a novel prognostic biomarker in colorectal cancer. Br J Cancer, 2015, 113(1): 83-90. doi: 10.1038/bjc.2015.168 26035698 PMC4647533

[42] Sun JY, Huang Y, Li JP, et al. MicroRNA-320a suppresses human colon cancer cell proliferation by directly targeting β-catenin. Biochem Biophys Res Commun, 2012, 420(4): 787-792. doi: 10.1016/j.bbrc.2012.03.075 22459450

[43] Zhang Q, Guan F, Fan T, et al. LncRNA WDFY3-AS2 suppresses proliferation and invasion in oesophageal squamous cell carcinoma by regulating miR-2355-5p/SOCS2 axis. J Cell Mol Med, 2020, 24(14): 8206-8220. doi: 10.1111/jcmm.15488 32536038 PMC7348145

[44] Yu L, Zhang W, Wang P, et al. LncRNA SNHG 11 aggravates cell proliferation and migration in triple-negative breast cancer via sponging miR-2355-5p and targeting CBX5. Exp Ther Med, 2021, 22(2): 892. doi: 10.3892/etm.2021.10324 PMC8243335 34257707

[45] Gong ZH, Ji J, Yao J, et al. SphK1-targeted miR-6784 inhibits functions of skin squamous cell carcinoma cells. Aging, 2021, 13(3): 3726-3741. doi: 10.18632/aging.202336 33465049 PMC7906188

[46] Shi L, Zhu W, Huang Y, et al. Cancer-associated fibroblast-derived exosomal microRNA-20a suppresses the PTEN/PI3K-AKT pathway to promote the progression and chemoresistance of non-small cell lung cancer. Clin Transl Med, 2022, 12(7): e989. doi: 10.1002/ctm2.989 PMC9299573 35857905

[47] Wei C, Dong X, Lu H, et al. LPCAT 1 promotes brain metastasis of lung adenocarcinoma by up-regulating PI3K/AKT/MYC pathway. J Exp Clin Cancer Res, 2019, 38(1): 95. doi: 10.1186/s13046-019-1092-4 PMC6385475 30791942

[48] Du X, Wang S, Liu X, et al. MiR-1307-5p targeting TRAF 3 upregulates the MAPK/NF-κB pathway and promotes lung adenocarcinoma proliferation. Cancer Cell Int, 2020, 20: 502. doi: 10.1186/s12935-020-01595-z 33061854 PMC7552495

[49] Lin HC, Li J, Cheng DD, et al. Nuclear export protein CSE1L interacts with P65 and promotes NSCLC growth via NF-κB/MAPK pathway. Mol Ther Oncolytics, 2021, 21: 23-36. doi: 10.1016/j.omto.2021.02.015 33869740 PMC8039531

[50] Tian Y, Zhang L, Yu Q, et al. MiR-135a inhibits non-small cell lung cancer progression by suppressing RAB1B expression and the RAS pathway. Aging, 2020, 12(14): 14480-14489. doi: 10.18632/aging.103494 32710726 PMC7425451

[51] Fu NJ, Xi RY, Shi XK, et al. Hexachlorophene, a selective SHP 2 inhibitor, suppresses proliferation and metastasis of KRAS-mutant NSCLC cells by inhibiting RAS/MEK/ERK and PI3K/AKT signaling pathways. Toxicol Appl Pharmacol, 2022, 441: 115988. doi: 10.1016/j.taap.2022.115988 35307375

[52] Wang Z, Huang Y, Lu W, et al. c-myc-mediated upregulation of NAT 10 facilitates tumor development via cell cycle regulation in non-small cell lung cancer. Med Oncol, 2022, 39(10): 140. doi: 10.1007/s12032-022-01736-6 35834140

[53] An Y, Xia Y, Wang Z, et al. Clinical significance of ribosome production factor 2 homolog in hepatocellular carcinoma. Clin Res Hepatol Gastroenterol, 2024, 48(3): 102289. doi: 10.1016/j.clinre.2024.102289 38307254

[54] Wu Z, Guo L, Wan L, et al. Comprehensive bioinformatics analysis of a RBM family-based prognostic signature with experiment validation in hepatocellular carcinoma. J Cancer Res Clin Oncol, 2023, 149(13): 11891-11905. doi: 10.1007/s00432-023-05084-4 37410140 PMC11797974

[55] Wang W, Zhang R, Feng N, et al. Overexpression of RBM34 promotes tumor progression and correlates with poor prognosis of hepatocellular carcinoma. J Clin Transl Hepatol, 2023, 11(2): 369-381. doi: 10.14218/JCTH.2022.00166 36643033 PMC9817046

[56] Yin Y, Zhou L, Zhan R, et al. Identification of WDR12 as a novel oncogene involved in hepatocellular carcinoma propagation. Cancer Manag Res, 2018, 10: 3985-3993. doi: 10.2147/CMAR.S176268 30310320 PMC6166768

[57] Dai G, Guo Z, Chen H, et al. High expression of guanine nucleotide-binding protein-like-3-like is associated with poor prognosis in esophageal cancer. Medicine, 2021, 100(21): e25993. doi: 10.1097/MD.0000000000025993. PMC8154413 34032716

[58] Hu DX, Sun QF, Xu L, et al. Knockdown of DEAD-box 51 inhibits tumor growth of esophageal squamous cell carcinoma via the PI3K/AKT pathway. World J Gastroenterol, 2022, 28(4): 464-478. doi: 10.3748/wjg.v28.i4.464 35125830 PMC8790558

[59] Fukawa T, Ono M, Matsuo T, et al. DDX31 regulates the p53-HDM2 pathway and rRNA gene transcription through its interaction with NPM1 in renal cell carcinomas. Cancer Res, 2012, 72(22): 5867-5877. doi: 10.1158/0008-5472.CAN-12-1645 23019224

[60] Han Z, Liu D, Chen L, et al. PNO 1 regulates autophagy and apoptosis of hepatocellular carcinoma via the MAPK signaling pathway. Cell Death Dis, 2021, 12(6): 552. doi: 10.1038/s41419-021-03837-y PMC8163843 34050137

[61] Li J, Xie S, Zhang B, et al. UTP 23 is a promising prognostic biomarker and is associated with immune infiltration in breast cancer. Crit Rev Eukaryot Gene Expr, 2024, 34(3): 1-15. doi: 10.1615/CritRevEukaryotGeneExpr.2023048311 38305284

[62] Zhao D, Qian L, Zhuang D, et al. Inhibition of ribosomal RNA processing 15 Homolog (RRP15), which is overexpressed in hepatocellular carcinoma, suppresses tumour growth via induction of senescence and apoptosis. Cancer Lett, 2021, 519: 315-327. doi: 10.1016/j.canlet.2021.07.046 34343634

[63] Zhou HZ, Li F, Cheng ST, et al. DDX17-regulated alternative splicing that produced an oncogenic isoform of PXN-AS 1 to promote HCC metastasis. Hepatology, 2022, 75(4): 847-865. doi: 10.1002/hep.32195 34626132 PMC9304246

